# Opposing effects of D-aspartic acid and nitric oxide on tuning of testosterone production in mallard testis during the reproductive cycle

**DOI:** 10.1186/1477-7827-6-28

**Published:** 2008-07-04

**Authors:** Maria M Di Fiore, Claudia Lamanna, Loredana Assisi, Virgilio Botte

**Affiliations:** 1 Department of Life Sciences, Second University of Naples, via Vivaldi 43, 81100 Caserta, Italy; 2 Department of Zoology, University of Naples 'Federico II', via Mezzocannone 8, 80134 Naples, Italy

## Abstract

**Background:**

D-Aspartic acid (D-Asp) and nitric oxide (NO) play an important role in tuning testosterone production in the gonads of male vertebrates. In particular, D-Asp promotes either the synthesis or the release of testosterone, whereas NO inhibits it. In this study, we have investigated for the first time in birds the putative effects of D-Asp and NO on testicular testosterone production in relation to two phases of the reproductive cycle of the adult captive wild-strain mallard (Anas platyrhynchos) drake. It is a typical seasonal breeder and its cycle consists of a short reproductive period (RP) in the spring (April-May) and a non reproductive period (NRP) in the summer (July), a time when the gonads are quiescent. The presence and the localization of D-Asp and NO in the testis and the trends of D-Asp, NO and testosterone levels were assessed during the main phases of the bird's reproductive cycle. Furthermore, in vitro experiments revealed the direct effect of exogenously administered D-Asp and NO on testosterone steroidogenesis.

**Methods:**

By using immunohistochemical (IHC) techniques, we studied the presence and the distributional pattern of D-Asp and NO in the testes of RP and NRP drakes. D-Asp levels were evaluated by an enzymatic method, whereas NO content, via nitrite, was assessed using biochemical measurements. Finally, immunoenzymatic techniques determined testicular testosterone levels.

**Results:**

IHC analyses revealed the presence of D-Asp and NO in Leydig cells. The distributional pattern of both molecules was in some way correlated to the steroidogenic pathway, which is involved in autocrine testosterone production. Indeed, whereas NO was present only during the NRP, D-Asp was almost exclusively present during the RP. Consistently, the high testosterone testicular content occurring during RP was coupled to a high D-Asp level and a low NO content in the gonad. By contrast, in sexually inactive drakes (NRP), the low testosterone content in the gonad was coupled to a low D-Asp content and to a relatively high NO level. Consequently, to determine the exogenous effects of the two amino acids on testosterone synthesis, we carried out in vitro experiments using testis sections deriving from both the RP and NRP. When testis slices were incubated for 60 or 120 min with D-Asp, testosterone was enhanced, whereas in the presence of L-Arg, a precursor of NO, it was inhibited.

**Conclusion:**

Our results provide new insights into the involvement of D-Asp and NO in testicular testosterone production in the adult captive wild-strain mallard drake. The localization of these two molecules in the Leydig cells in different periods of the reproductive cycle demonstrates that they play a potential role in regulating local testosterone production.

## Background

It has been long known that testicular testosterone synthesis in vertebrates occurs in Leydig cells after LH stimulation [1]. In the last few decades, however, several studies have pointed out that in some laboratory mammals, besides the substantial role of LH [1], a variety of other local biochemical agents are also accountable for testosterone production. Some of these agents, acting either as stimulators or as inhibitors of testosterone production, have been localized in Leydig cells and in a variety of neighboring cells – including Sertoli cells and leucocytes – where they act as autocrine [2], paracrine [3,4] or endocrine hormones [5,6].

Among the array of regulatory agents so far discovered, two molecules, *viz*, the amino acid D- aspartate (D-Asp) and the inorganic free radical gas nitric oxide (NO), have lately drawn our attention. These two small molecules belong to the amino acid matrix generating system. Indeed, whereas D-Asp is the enantiomeric form of L-aspartic acid (L-Asp) resulting from its conversion *via *an aspartate racemase, NO is synthesized from L-arginine (L-Arg) *via *NO synthase (NOS).

Regarding their endocrine role, in the adult rat testis D-Asp operates on Leydig cell steroidogenic pathway by favoring testosterone synthesis [7-12], whereas NO appears to inhibit it [3,13-15]. These findings agree with a recent study showing that in boar testis testosterone production *in vitro *is stimulated by D-Asp and inhibited by an NO generating system [16].

In addition to these previous studies on mammals, a clear definition of the dynamic relationships between these regulatory factors and testicular testosterone production has also been obtained by studying seasonally breeding vertebrates, since in these animals the androgen synthesis is usually restricted to a short reproductive period of the annual sexual cycle. Indeed, in the green frog *Rana esculenta *[17] and in the lizard *Podarcis sicula *[18], highest testosterone production occurs in the springtime breeding period, a time when D-Asp concentration levels rise in the animals' testes. Moreover, it has been reported that when exogenous D-Asp is administered to sexually inactive (non reproductive) adult males, it temporarily induces a significant increase in testosterone output [17,18]. This field of research, however, has not yet been investigated in birds.

Hence, on the basis of this insightful research, we have investigated the trends of D-Asp and NO levels, together with testosterone content, in the testes of a typical seasonal breeder, the duck *Anas platyrhynchos*, whose male gonads produce both gametes and sex hormones almost exclusively during the spring [19,20]. Therefore, we determined D-Asp, NO, and testosterone concentrations in the testes of reproductive (RP) and non reproductive (NRP) adult male ducks. Furthermore, since the intratesticular source of both D-Asp and NO is still poorly defined, we also investigated the putative storage and/or production sites of these substances by immunohistochemical analysis. Finally, experiments *in vitro *revealed the direct effect of exogenously administered D-Asp and NO on testosterone steroidogenesis.

We hope that the results of the present study, in addition to having deepened our understanding of the complex mechanisms involved in the reproductive system of vertebrates, may also make a significant contribution to the already enlightening data on breeding biology.

## Methods

### Reproductive aspects of the sexual cycle in male Anas platyrhynchos

The annual reproductive cycles in male mallards have been well characterized [20-22] on the basis of studies which have compared the relationship between reproductive features and blood testosterone levels in drakes. In mallards exposed to natural lighting, the breeding season is restricted to the spring. During winter, birds become photosensitive and in this state respond to increasing photoperiods with gonadal growth and increasing plasma testosterone levels [22]. When the breeding season begins, *ie*, in April and May [21,23], circulating testosterone reaches peak concentrations and the testes become fully developed for the reproductive activity. On the contrary, in the late spring and early summer, birds become photorefractory, as the reproductive activity cannot be maintained throughout the lengthy daylight hours accompanying this season. This response gives rise to a rapid decline in circulating concentrations of testosterone and regression of the testes [23]. The decline in plasma concentrations of testosterone is correlated with the postnuptial molt at the end of which drakes are rendered flightless for 2–3 weeks owing to the loss of their primary flight feathers [24]. After the postnuptial molt, the drake can no longer be distinguished from the hen on the basis of plumage color, as the drake's characteristic green head feathers are replaced by brown feathers. When the growth of new flight feathers is completed, the prenuptial molt begins [24]. The testes appear to be completely regressed by the time the complete prenuptial plumage is acquired [24] and the drakes have returned to their characteristic breeding plumage. A second peak in testosterone occurs in the late fall, but unlike the first, it is not accompanied by an increase in testicular size; rather, it is thought to be related to pair bond formation [21].

### Animal compliance

Adult male ducks, *Anas platyrhynchos*, weighing 2.5–3.5 Kg, were obtained from a farmer during their reproductive (RP) and non reproductive periods (NRP). The collection of samples for the RP was done between April 29^th ^and May 2^nd ^of 2007. All drakes were in nuptial plumage and no signs of molt were visible. Therefore, all males were considered to be in their breeding condition. The collection of samples for the NRP was done in July (15–20, 2007) when the animals showed the typical feature of post-nuptial molt and the characteristic green head feathers had been replaced by brown feathers.

Six specimens for each period were utilized. The birds were first anesthetized by an intramuscular injection of ketamine chloridate (25 mg/kg), and, then sacrificed. The methods of dissection were in accordance with the Italian guidelines for animal handling (D. L.vo 116/92) and authorized by the local Animal Care Committee (Servizio Veterinario della A.S.L. 44, Prot. Vet. 22/95).

The testes were rapidly dissected out. For each testis, three samples were immediately frozen in liquid nitrogen for biochemical determination of D-Asp, NO and testosterone. Other samples were first fixed in Bouin's fluid for 12–24 h at room temperature. Then they were dehydrated, embedded in Paraplast and cut on microtome into 7 μm sections for the routine histology and immunohistochemical staining of D-Asp and NO. In addition, fresh testes were utilized for *in vitro *tests (see below).

### Immunohistochemistry

To localize D-Asp and NO in the testes, five randomly chosen sections for each duck testis were used. The avidin-biotin complex (ABC) (Vector Laboratories, PK-6101) was used for immunohistochemical analysis. Polyclonal antibodies, raised in rabbits and directed against D-Asp (Inst. Bas. Sci., Oslo) and nNOS (S. Cruz Biotechnol., SC 648), were diluted at 1:400 and applied to the sections which had previously been incubated overnight in a humid chamber at 4°C. The secondary antiserum, raised in goat, and the PAP complex were applied to the sections for 30 min at room temperature. The sites on the immunoreactive reaction were amplified using an H_2_O_2 _activated solution either of 3,3' diaminobenzidine (DAB) (Vector Laboratories, SK 4100) or of complex DAB-nichel (Vector Laboratories, SK 4100). Sections were counterstained with hematoxylin, dehydrated in ethanol, cleared in xylene, and mounted.

The specificity of immunostaining for both D-Asp and NO was determined by 1) the substitution of the primary antiserum with 0.1 M PBS or normal rabbit serum and 2) the overnight pre-absorption of the primary antiserum with synthetic antigen (for both D-Asp and NOS, Sigma). These controls never showed any positive reaction.

The preparations were revealed by Nikon E 600 light microscope and microphotographs were taken by Kodak Tech Pan film.

The scoring of immunoreaction was performed on five randomly chosen sections from each animal testis on the total of five different fields for each slide.

### Morphometry

To test the putative differences of immunopositive cells for D-Asp and NO synthase, we performed a morphometric analysis using an image analyzer system. Five randomly chosen sections of testis (D-Asp or NOS immunostained) for each animal of each reproductive stage were viewed at a magnification of ×1000. The morphological parameter measured was the number of the immunoreactive Leydig cells in the testis in 1 mm^2^. Morphometric analysis consisted of digitization of transverse sections viewed under a Nikon Eclipse E600 light microscope with an attached JVCTK-C1381 photocamera connected to a Penthium II computer running Lucia ScMeas on Mutech software.

**Figure 1:**
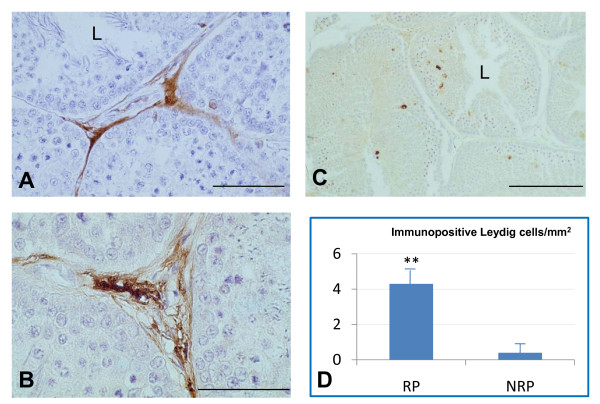
**Representative immunohistochemical distribution and localization of D-Asp in the testes of the duck *A. platyrhynchos *during the reproductive period (RP) (**A **and **B**) and the non reproductive period (NRP)** (**C**). D-Asp immunopositivity was mainly localized in Leydig cells of the RP (**A **and **B**) and completely absent in the NRP (**C**). Histograms represent quantification of immunopositive Leydig cells/mm^2 ^(**D**). **, p < 0.01 RP versus NRP. L, lumen. Scale bars: a, 50 μm; b, 20 μm; c, 100 μm.

**Figure 2:**
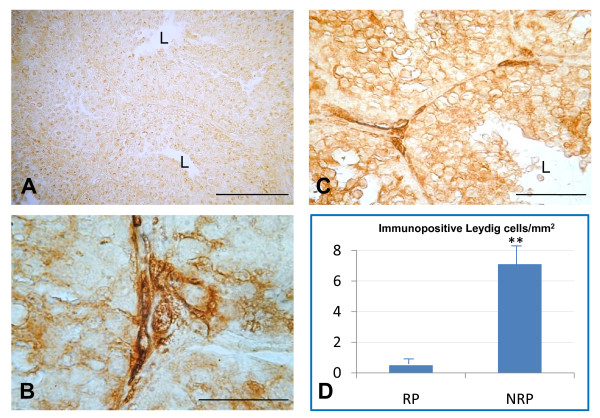
**Representative immunohistochemical distribution and localization of NO synthase in the testes of duck *A. platyrhynchos *during the reproductive period (RP) (**A**) and the non reproductive period (NRP) (**B **and **C**).** NOS positive cytotypes were found elsewhere in seminiferous tubules, but they were very rare in the interstitial tissue of RP (**A**). Immunopositivity was mainly localized in Leydig cells of the NRP (**B **and **C**). Histograms represent quantification of immunopositive Leydig cells/mm^2 ^(**D**). **, p < 0.01 NRP versus RP. L, lumen. Scale bars: a, 100 μm; b, 50 μm; c, 20 μm.

Immunopositive Leydig cells for both D-Asp and NOS between the two reproductive periods are presented in the form of histograms in Figures 1 and 2, respectively.

### Quantitative D-Asp determination

Frozen tissue samples were thawed and homogenized with 0.5 M perchloric acid (PCA) in a 1:10 ratio and centrifuged at 30,000 g for 20 min. Supernatants, brought to pH 7.5–8.5 with 5 M KOH, were cooled for 30 min at 0°C. Potassium perchlorate precipitate was removed by centrifugation. Supernatants were adjusted to a pH of about 2.5 with 1 M HCl, and the amino acids were purified on a cation exchange column (AG 50W-X8 resin, hydrogen ionic form, 200–400 mesh, Bio-Rad). Samples were loaded onto columns (1 × 3 cm) equilibrated with 0.01 M HCl, and, after a wash with 10 ml 0.01 M HCl, they were eluted with 8 ml of 4 M NH_4_OH. The elutes were dried by evaporation in small Petri dishes on a hot plate at 40–60°C under a hood. The dried elutes were dissolved in 1 ml of 0.01 M HCl. They were then purified by slowly passing them, by means of a syringe, through a Sep-Pak C-18 cartridge (300 mg; Waters, Milan, Italy) which had been previously activated with methanol or acetonitrile and washed with distilled water. In order to recover all the amino acids from these elutes, the cartridge was eluted twice with 2 ml of 0.01 M HCl. The resulting elutes were combined and either dried using a Savant centrifuge or left to evaporate in small Petri dishes at 40–50°C under the hood. The dry residues were then dissolved in 200 μl 0.01 M HCl and analyzed for D-Asp content. The assay procedure was identical to that previously described by Di Fiore et al. [25]. A standard curve was obtained by applying the enzymatic method to D-Asp solutions of known concentrations.

### Biochemical measurement of NO activity

NO was assessed according to Meli et al. [26] by measuring tissue nitrite, an indicator of NO synthesis. Briefly, frozen samples of testes were thawed and rinsed in cold PBS buffer (0.1 M, pH 7.4). Then they were weighed and homogenized (1.2, w/v) in the same ice-cold buffer. The suspensions were centrifuged at 3500 g for 15 min and aliquots of 100 μl of supernatant were mixed with aliquots of 100 μl of Griess reagent containing 1% sulphanilamide and 0.1% naphthlethylenediamine in 5% phosphoric acid. The suspensions were first kept at room temperature for 10 min and then their absorbance (550 nm) was measured in a micro-plate reader (Titertek multiskan MCC). Solutions of sodium nitrite were used as standards. Nitrite concentrations in tissues were calculated by comparing their OD with those of the standard solutions. The results were expressed as mmol of nitrite (NO_2_^-^) released by one g of fresh tissue.

### Testosterone determination

Determination of testosterone levels in the testis extracts was carried out using an enzyme immunoassay kit (EIA) (Adaltis, Bologna, Italy). The following limits of detection were observed for testosterone: sensitivity 6 pg, intra-assay variability 5.3 %, inter-assay variability 7.5 %. Testis samples were homogenized in the ratio of 1:10 (w/v) with distilled water. The homogenate was then mixed vigorously with ethyl ether (1:10, v/v) and the ether phase was withdrawn after centrifugation at 3000 g for 10 min. Three extractions were performed. Pooled ether extracts were dried by evaporation and the residue was dissolved in a 0.5 ml sodium phosphate buffer 0.05 M (pH 7.5), containing BSA at a concentration of 10 mg/ml. Finally, it was utilized for testosterone immunoassay, as previously reported [25]. The rate of testosterone recovery from testis was about 80%.

### In vitro tests

Testes were minced and aliquots (500 mg each) were distributed into a multi-well plate with 3 ml of KRB buffer (Krebs-Ringer bicarbonate buffer (pH 7.4) containing 10 mM glucose, 100 μM bacitracin, 0.1% ascorbic acid, and 0.1% bovine serum albumin). The samples were preincubated for 60 min in an atmosphere of 95% 0_2_/5% CO_2 _with constant shaking at 60 cycles per min at 37°C. Then the medium was substituted with 3 ml of fresh KRB buffer containing increasing concentrations of either D-aspartic acid (D-Asp) in a range between 0.5–2.0 mM or L-arginine (L-Arg) in a range between 1.0–2.0 mM (Sigma). The samples were finally incubated for 60 and 120 min. Controls were incubated in medium alone. At the end of the incubation period, the media were aspirated and frozen. Testosterone was measured as reported above. The results were expressed in terms of nanograms (ng) of testosterone released per gram (g) of tissue. The experiments were performed in triplicate and data were expressed as means ± SD. Statistically significant differences were set at p < 0.05 and p < 0.01.

### Statistical analysis

Data were compared by analysis of variance followed by Duncan's test for multi-group comparison and Student *t *test for between-group comparison. All data were expressed as mean ± standard deviations. The level of significance was taken at P < 0.01 and P < 0.05.

## Results

### Presence and distribution of D-Asp and NOS

In the examined ducks, the testes had the typical high vertebrate texture with a tight network of seminiferous tubules, interposed with interstitial cells, *ie *Leydig cells. These cells were usually well distinguishable because of their larger volume and roundish shape. In the intertubular spaces, they were isolated or grouped in clusters with a variable number of components. The morphometric analysis revealed no statistical difference (p > 0.05) in the number of Leydig cells expressed during the two distinct phases of reproductive cycle.

In the testis, seminiferous tubules showed active spermatogenesis only in the reproductive ducks when Sertoli cells were more visible.

The immunohistochemical tests detected D-Asp and NOS positive materials mostly in Leydig cells. A feeble positive reaction was also noticed in Sertoli cells, in peritubular cells surrounding the seminiferous tubule, in spermatogonia and in spermatids. No differences in their staining pattern were observed during either period (description not given).

Differences in the number of Leydig cells involved and in their staining intensity to D-Asp and NOS immunoreactive methods were observed in animals deriving from both phases of reproductive cycle. Indeed, whereas NOS staining intensity was mainly greater during NRP, D-Asp immunoreactivity was greater during RP.

Figure 1 reports D-Asp immunoreactive distribution in testes of ducks belonging to the RP (Figure. 1A and 1B) and to the NRP (Figure. 1C). D-Asp immunoreactivity was primarily found in Leydig cells (Figure. 1A and 1B). The positive Leydig cells were more numerous and mainly grouped in clusters during the RP (Figure. 1A). Figure 1B shows a section at higher magnification of immunopositive Leydig cell types. During the NRP (Figure. 1C), reactive Leydig cells were present only in few of the examined sections and, when present, they appeared isolated. The morphometric analysis (Figure. 1D) revealed the quantification of immunopositive Leydig cells/mm^2 ^during both periods: immunopositivity was higher during the RP than during the NRP (p < 0.01).

NOS immunoreactive distribution in the testes of ducks belonging to the RP (Figure. 2A) and to the NRP (Figure. 2B and 2C) is shown in Figure. 2. In the latter, NOS-positive Leydig cells were always present and the finding of interstitial spaces entirely filled with reactive Leydig cells was common. Figure 2B shows a section at higher magnification of immunopositive Leydig cell types. Figure 2A shows a sample section of the RP, where the immunopositive Leydig cell types were weak. Figure 2D shows the histograms of the quantification of immunopositive Leydig cells/mm^2 ^during both periods: they were higher during the NRP than during the RP (p < 0.01).

### Determination of testicular D-Asp, NO activity and testosterone

**Figure 3:**
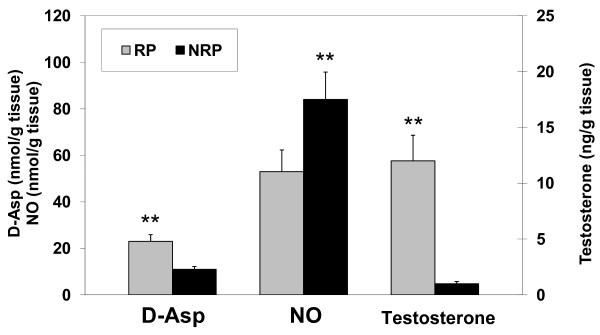
**Concentrations of D-Asp, NO levels and titers of testosterone in the testes of *A. platyrhynchos *during the reproductive period (RP) and non reproductive period (NRP).** D-Asp concentration was higher in RP, corresponding to maximum testosterone titre and to minimum NO level. Each point represents the mean value ± S.D. from six individual ducks. **, p < 0.01, Reproductive versus Non Reproductive period.

Figure 3 reports the levels of D-Asp, NO and testosterone in the duck testicular extracts of the RP and the NRP. Biochemical analysis revealed that D-Asp concentration in duck testis was higher during the RP than during the NRP (23.1 ± 2.9 nmol/gr of wet tissue and 11.0 ± 1.2 nmol/gr of wet tissue, respectively), (p < 0.01).

The biochemical measurement of NO_2_^- ^tissue content performed by Meli's method revealed 53.2 ± 9.3 nmol/gr during RP, and 84.7 ± 11.8 nmol/gr during the NRP (p < 0.01). Testosterone level in testicular extracts was 12.5 ± 2.3 ng/g tissue in RP and 0.87 ± 0.2 ng/g tissue in NRP (p < 0.01).

### In vitro effect of D-Asp and L-Arg on testosterone release

**Figure 4:**
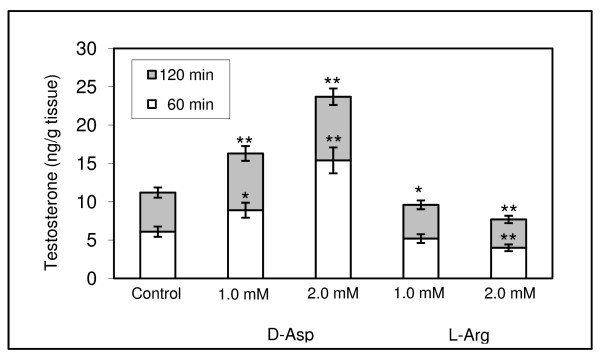
**Effect of increasing concentrations of D-Asp (1.0–2.0 mM) and of L-Arg (1.0–2.0 mM) on testosterone release from testes of *A. platyrhynchos*.** The testis slices were incubated for 60 (□) or 120 (■) min. Testosterone levels were enhanced after 60 or 120 min incubation with D-Asp, whereas they were inhibited in the presence of L-Arg. The level of significance was taken at p < 0.05 (*) and p < 0.01 (* *) versus respective controls.

To verify the exogenous effects of D-Asp and L-Arg, a precursor of NO, on testicular testosterone release, we incubated slices of duck testes for 60 and 120 min in a medium containing either D-Asp or L-Arg (Figure 4). As expected, D-Asp induced a significant increase in testosterone, whereas L-Arg caused a decrease. Specifically, after a 60-min incubation with D-Asp, testosterone synthesis was higher than that of control (2.0 mM [p < 0.01] and 1.0 mM [p < 0.05]). Similarly, after 120 minutes of incubation, the increases were significantly higher than those of control (1.0 mM and 2.0 mM D-Asp concentration [p < 0.01]). Furthermore, when the exogenous stimulation was absent, we noted that testosterone production at 120 min was approximately 2-fold higher than that at 60 min. In fact, although a 60-min incubation with D-Asp yielded higher testosterone values, the proportionate increase at 120 min appeared approximately identical to that of control.

Incubations with L-Arg yielded a decrease in testosterone levels after 120 min at 1.0 mM (p < 0.05) and after 60 and 120 min of incubation at 2.0 mM (p < 0.01), as compared to controls.

## Discussion

Since the 1950s, Leydig cells have been identified as the androgen source in vertebrate testes, and, thereafter, in the late 1960s and early 1970s, their endocrine activity was shown to be strictly dependent on the pituitary LH [1]. In the last decades, however, new technical advances have made it possible to delve deeper into the intricate mechanisms underlying the regulation of steroidogenesis, particularly in mammalian testes. These studies have soon shown that a myriad of intratesticular agents present in Leydig cells can alter testosterone production by acting as autocrine [2], paracrine [3,4], and endocrine [5,6] excitatory or inhibitory factors.

In recent years, several studies in this burgeoning filed of research have attributed a great importance to the regulating properties of two molecules, *viz*, the amino acid D-aspartic acid (D-Asp) and the free radical Nitrogen Oxide (NO) in the reproductive systems of mammals [27,28].

However, the putative role of D-Asp and NO in testosterone production in the male gonads of some seasonal breeder vertebrates also offers a good basic unmanipulated model to improve our understanding of reproductive biology. In these animals, the gonads are active only for a relatively short period each year, specifically in the spring. During this season, sex hormone plasma concentrations peak, gametes are produced and the secondary sexual characters (SSC) completely develop. By contrast, sex hormone production is very low throughout the rest of the year.

In the present study we report for the first time the involvement of D-Asp and NO in the testis steroidogenesis of the mallard *A. platyrhynchos*, a typical seasonal breeder. Such hypothesis was verified by assaying testicular testosterone production in relation to the presence and distribution of D-Asp and NO during the reproductive period (RP) and the non reproductive period (NRP). Moreover, to better evaluate the role of exogenous D-Asp and NO in testicular testosterone synthesis, *in vitro *experiments were carried out.

Immunocytological techniques have allowed scientists to localize D-Asp in Leydig and Sertoli cells [7], as well as in peritubular and several germinal epithelium elements [29]. A strong correlation between D-Asp content and testosterone synthesis has been found in the testes of rats [7-12], boar [16], amphibians [17], and reptiles [18], where, indeed, D-Asp stimulates testosterone synthesis and release. In Leydig cells, D-Asp appears to act on genes since it induces the synthesis of the regulatory protein StAr involved in the synthesis of testosterone [11]. Consistently, in the present study we found that free D-Asp was endogenously contained in the mallard testis and varied throughout the two sexual reproductive cycles, *ie *the reproductive period (RP) and non reproductive period (NRP). D-Asp concentration was higher during the RP (23.9 ± 2.9 nmol/g tissue) than during the NRP (11.0 ± 1.2 nmol/g tissue). Such discrepancy was also evident in IHC studies, in which, although no difference in the number of Leydig cells was found between the two phases of the reproductive cycle considered, a significant increase in D-Asp immunopositive Leydig cells was detected during the RP but not during the NRP.

Circumstantial evidence suggests that Nitric Oxide (NO) is normally synthesized in the mammalian testis. The enzyme nitric oxide synthase (NOS), which determines NO formation in tissues through the oxidation of L-arginine, has indeed been localized in several testicular components, including Leydig and Sertoli cells [13,16,30-33]. Intriguingly, our immunohistochemical observations only in part support such theories, for we found that NO was mostly localized in Leydig cells. Intriguingly, here we report for the first time that NO is far more expressed during the NRP than during the RP. Indeed, our morphometric analysis revealed a more intense NO-immunopositivity during the NRP, as compared to the RP. Moreover, NO concentration resulted inversely correlated to either D-Asp or testosterone in the two periods of the reproductive cycle. In fact, the highest NO concentrations were registered in the testes during the NRP (84.7 ± 11.8 nmol/g tissue). Interestingly, this increase was paralleled by minimum testosterone levels (0.87 ± 0.2 ng/g tissue) and by the regression of the testes. Thus considering that during this period the animal undergoes postnuptial molt [21], we could advance the hypothesis that highest levels of NO are somehow related to molt. Indeed, a recent study done on *Gallus domesticus *has emphasized that NO production increases after molt induction [34]. Nonetheless, further studies are still required to validate this hypothesis in mallards.

In current literature, various functions have been ascribed to NO in the testis, such as the stimulation of germ cell metabolism/apoptosis, the relaxation of vascular myocytes, the regulation of peristalsis and permeability of the seminiferous tubule [28], the inhibition of sperm motility, the induction of peritubular myofibroblast contraction [14,31,35-39], and, lastly, the significant depression of testosterone synthesis in Leydig cells. The latter mechanism, however, is still poorly defined. So far, it has been speculated that NO could inhibit Leydig cell testosterone synthesis either directly or indirectly. More specifically, when NO is released within the testes by either Leydig cells or by intraorganic nerve fibres [32], it could activate soluble guanylyl cyclase (sGC) and, consequently, cyclic guanosin monophosphate (cGMP) [30,38]. The latter, in turn, could induce the stimulation of a yet poorly defined cytoplasmic protein kinase [40]. It is worthy of note that the sGC/cGMP pathway of cellular response to NO has been proposed not only for the Leydig cell but also for other testicular cytotypes including Sertoli, endothelial, peritubular, and germ cells [41].

Even though the literature on NO underscores its direct or indirect role in the regulation of gonadal functions, specific research on avian species is still limited. Only recently, in an interesting study carried out on Japanese quails, it has been suggested that NO plays a positive regulatory role in the physiology of gonadal and adrenal axis and photosexual responses [42]. Remarkably, however, the data that we report in this study, despite appearing inconsistent with current findings, actually do highlight two different aspects of analysis with respect to NO involvement. For instance, although the study on sexually immature Japanese quails pinpoints several factors that can enhance the regulation of hypothalamo-hypophyseal-gonadal and -adrenal axis, it does not mention how NO could possibly regulate steroidogenesis in Leydig cells. These discrepancies could be ascribable to the differences among species, in particular with regard to their status of sexual maturity and/or regulation machinery considered.

Moreover, what makes this study particularly insightful is the evidence that both D-Asp and NO displayed a local and direct action on Leydig cells that was strictly related to the two different phases of the sexual cycle. Indeed, it emerged that the two phases of the reproductive cycle were characterized by different biochemical pathways. More specifically, we observed that during the spring, whereas NO content was relatively low, highest concentrations of testosterone and D-Asp occurred in the testes of sexually active drakes. Conversely, in the testes of non reproductive males, testosterone and D-Asp levels declined markedly, whereas NO potential synthesis was relatively high. These intriguing findings were further corroborated by parallel results obtained from our *in vitro *experiments. The testosterone release from testis slices of reproductive ducks was stimulated when we added exogenous D-Asp to the incubation medium. By contrast, it was inhibited when we incubated the sections with an NO generating system.

In conclusion, these results provide substantial support to the hypothesis that D-Asp and NO play important regulatory roles in testicular testosterone production. Thus, given their intrinsic capability to balance hormonal production in Leydig cells, they ought to be considered putative opposite regulators of local testosterone synthesis. Equally important, our findings suggest that these factors could also be implicated in Leydig cell endocrine activity, as it occurs in seasonally reproducing vertebrates. Finally, we hope that the results of the present study, in addition to having deepened our understanding of the complex mechanisms involved in the reproductive system of vertebrates, may also make a significant contribution to the already enlightening data on breeding biology.

## Competing interests

The authors declare that they have no competing interests.

## Authors' contributions

MMDF devised the study, organized the planning of the experiments, developed the method for D-Asp determination, performed *in vitro *experiments and wrote the manuscript. CL carried out the sampling during the reproductive stages, developed the IHC methods and NO assays, took part in the discussion of the results and helped write manuscript. LA measured testosterone levels, made statistical analyses, took part in the discussion of the results. VB participated in the discussion of the results and helped write the manuscript. All authors read and approved the final manuscript.
